# Antifungal susceptibility profile of *Candida* clinical isolates from 22 hospitals of São Paulo State, Brazil

**DOI:** 10.1590/1414-431X2020e10928

**Published:** 2021-06-14

**Authors:** D.K.B. Rodrigues, L.X. Bonfietti, R.A. Garcia, M.R. Araujo, J.S. Rodrigues, V.M.F. Gimenes, M.S.C. Melhem

**Affiliations:** 1 Núcleo de Micologia do Instituto Adolfo Lutz, Secretaria de Saúde, São Paulo, SP, Brasil; 2 Laboratório de Micologia Médica-LIM 53, Instituto de Medicina Tropical, Universidade de São Paulo, São Paulo, SP, Brasil; 3 Escola de Medicina, Universidade Federal do Mato Grosso do Sul, Mato Grosso do Sul, MS, Brasil

**Keywords:** Candidemia, Drug resistance, Candida haemulonii, Candida glabrata, Echinocandins

## Abstract

This study aimed to evaluate the frequency of cryptic *Candida* species from candidemia cases in 22 public hospitals in São Paulo State, Brazil, and their antifungal susceptibility profiles. During 2017 and 2018, 144 isolates were molecularly identified as 14 species; *C. parapsilosis* (32.6%), *C. albicans* (27.7%), *C. tropicalis* (14.6%), *C. glabrata* (9.7%), *C. krusei* (2.8%), *C. orthopsilosis* (2.8%), C. *haemulonii var. vulnera* (2.1%), *C. haemulonii* (1.4%), *C. metapsilosis* (1.4%), *C. dubliniensis* (1.4%), *C. guilliermondii* (1.4%), *C. duobushaemulonii* (0.7%), *C. kefyr* (0.7%), and *C. pelliculosa* (0.7%). Poor susceptibility to fluconazole was identified in 6.4% of *C. parapsilosis* isolates (0.12 to >64 µg/mL), 50% of *C. guilliermondii* (64 µg/mL), 66.6% of *C. haemulonii var. vulnera* (16-32 µg/mL), and *C. duobushaemulonii* strain (MIC 64 µg/mL). Our results corroborated the emergence of *C. glabrata* in Brazilian cases of candidemia as previously reported. Importantly, we observed a large proportion of non-wild type *C. glabrata* isolates to voriconazole (28.6%; <0.015 to 4 µg/mL) all of which were also resistant to fluconazole (28.6%). Of note, *C. haemulonii*, a multidrug resistant species, has emerged in the Southeast region of Brazil. Our findings suggested a possible epidemiologic change in the region with an increase in fluconazole-resistant species causing candidemia. We stress the relevance of routine accurate identification to properly manage therapy and monitor epidemiologic trends.

## Introduction

*Candida* is a genus of yeasts that most frequently cause fungal bloodstream infections, known as candidemia, widely diffused in intensive care units, characterizing a life-threatening condition in critically ill patients (1). Candidemia cases are rising and candidemia is becoming a public health problem with a high financial burden, especially in developing countries (1).

The epidemiology of candidemia may vary according to geography, patient characteristics, and hospital system. Some risk factors associated with candidemia are the use of immunosuppressive drugs, antibiotics, and central venous catheters, especially in patients with hematologic diseases, neutropenia, and diabetes mellitus (2). In Brazil, the mortality rate of candidemia cases can be over 50%, higher than countries in Europe and North America (3).

Although *C. albicans* is the predominant species, studies have noted an increase of non-*albicans Candida* species. Molecular identification tools allow the identification of new cryptic species within larger species complexes, e.g., *C. albicans*, *C. parapsilosis*, *C. glabrata*, C. *haemulonii*, and *Meyerozyma guilliermondii* (*Candida guilliermondii*) (4). Understanding the distribution of cryptic species can help in the clinical management because some species show reduced susceptibility to triazoles or echinocandins (1).

Due to the large size, variable climate, and social-economical variability of regions in Brazil, there are relatively few representative data describing the etiologic agents of candidemia down to cryptic species in Brazil, or their susceptibility to antifungal drugs. We add new information on the cryptic species distribution and resistance occurrence among Brazilian isolates causing candidemia.

## Material and Methods

We collected 144 yeast isolates from individual patients that were submitted to the Reference State Laboratory Institute Adolfo Lutz between 2017 and 2018, from 22 public hospitals, including tertiary general hospitals (54.5%), teaching hospitals (40.9%), and infectious diseases hospitals (4.5%) distributed across 14 cities in São Paulo State, Brazil, as part of the National Antimicrobial Resistance Surveillance. The isolates were initially recovered from glycerol stock at -20°C and were subcultured onto CHROMagar™ *Candida* medium (Difco, USA) to evaluate colony purity and viability. Standard phenotypic methods such as microscopic morphology on corn meal agar plus Tween 80, growth at 37°C and 42°C, assessment of growth on 19 carbon and 2 nitrogen sources, fermentation of six carbohydrates, and morphological features were used to initially assign species (5).

### Molecular identification

For molecular identification of isolates initially identified as *C. albicans*, *C. glabrata,* and *C. parapsilosis*, we used PCR (polymerase chain reaction), multiplex PCR, and PCR-RFLP (polymerase chain reaction-restriction fragment length), respectively, with specific primers following previously published protocols (6–8). The DNA extraction was performed as described by Green and Sambrook (9) and concentration and purity were checked using NanoDrop 1000 (Thermo Fisher Scientific, USA). Additionally, matrix-assisted laser desorption/ionization-time of flight mass spectrometry (MALDI-TOF MS]-based identification of *C. haemulonii* and C. *guilliermondii* species complex isolates was performed using the ethanol/formic acid extraction protocol provided by the manufacturer (Bruker Daltonics, USA).

### Antifungal susceptibility testing

The minimum inhibitory concentrations (MICs) of antifungal agents for all of the isolates were determined by the reference broth microdilution method according to Clinical and Laboratory Standards Institute (CLSI) M27-ed4 (10) for fluconazole (FCZ), voriconazole (VCZ), caspofungin (CAS), anidulafungin (AND), micafungin (MICA), and amphotericin B (AmB). As a quality control, *C. parapsilosis* ATCC 22019 and *C. krusei* ATCC 6258 were used. MIC breakpoints were interpreted according to CLSI document M60 (11). When breakpoints were not available, the epidemiological cutoff values (ECVs) were employed to classify the isolates (12,13) (Supplementary Table S1). The ECVs allowed the differentiation between wild type and non-wild type isolates. Some species remain without breakpoints or ECVs for antifungals.

## Results

The 144 non-duplicate isolates were obtained from hemocultures coming from public hospitals that included regular hospitals (54.5%), teaching hospitals (40.9%), and hospitals specializing in infectious diseases (4.5%) located in 14 cities in São Paulo State, the most populous state in Brazil. The *C. parapsilosis* complex represented 36.8% (n=53) of the total species, of which *C. parapsilosis sensu stricto* (s.s.) accounted for 88.7% (n=47), *C. orthopsilosis* 7.5% (n=4), and *C. metapsilosis* 3.8% (n=2). The *C. albicans* complex was the second most frequent, representing 29.1% (n=42) of the total species with the following species *C. albicans* s.s. 95.2% (n=40) and *C. dubliniensis* 4.8% (n=2). No cryptic species were found for the *C. grabrata* complex, so the species *C. glabrata* s.s. was found with a frequency of 9.7% (n=14). The *C. haemulonii* complex represented 4.2% (n=6) of the candidemia cases. Within the complex, the following frequencies were found: *C. haemulonii* s.s. 33.3% (n=2), *C. haemulonii var. vulnera* 50% (n=3), and *C. duobushaemulonii* 16.66% (n=1). The frequency for *C. guilliermondii* s.s. was 1.2% (n=2) and no cryptic species were found. The frequencies of other species not belonging to a species complex were: *C. tropicalis* 14.6% (n=21), *C. krusei* 2.8% (n=4), *C. kefyr* 0.7% (n=1), and *C. pelliculosa* 0.7% (n=1).

**Figure 1:**
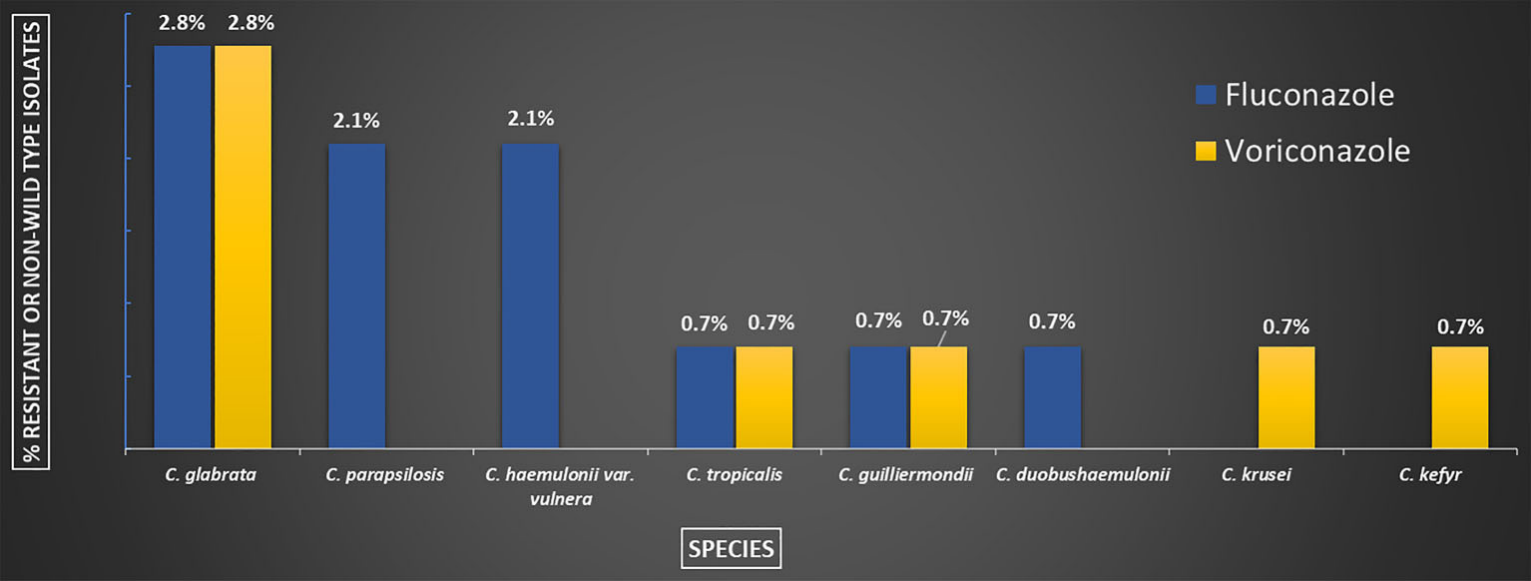
Percentage of fluconazole (n=13) and voriconazole (n=8) resistant, non-wild type or with high minimum inhibitory concentrations for 144 bloodstream *Candida* isolates, from hospitals in São Paulo, Brazil (2017-2018).

The MIC_50_ and MIC range of the susceptibility testing are summarized in Supplementary Table S2. Most of the isolates were susceptible or wild type to the tested antifungals. For amphotericin B, high MICs for *C. duobushaemulonii* were encountered. We found resistance or non-wild type to fluconazole (FCZ) in *C. parapsilosis* s.s. (6.4%; 3/47), *C. glabrata* s.s. (28.6%; 4/14), *C. guilliermondii* s.s. (50%; 1/2), and high MICs to *C. haemulonii var. vulnera* (100%; 3/3) and for the single strain of *C. duobushaemulonii.* For voriconazole (VCZ), resistance or non-wild type strains were observed among *C*. *krusei* (25%; 1/4), *C. guilliermondii* s.s. (50%; 1/2), and *C. glabrata* s.s. (28.6%; 4/14) isolates. Four strains of *C. glabrata* s.s. (28.6%; 4/14), one of *C. tropicalis* (4.8%; 1/21), and one of *C. guilliermondii* s.s. (50%; 1/2) exhibited high MICs for both FCZ and VCZ. The only strain of *C. kefyr* was non-wild type for CAS and VCZ and one isolate of *C. dubliniensis* was non-wild type for CAS. The percentage of resistant or non-wild type strains to fluconazole and voriconazole are summarized in Figure 1.

## Discussion

Antifungal susceptibility testing and the use of molecular biology are important tools not only as a subsidy for therapeutic guidance but also for monitoring the trend in the occurrence of species and resistance to antifungal agents over time in a given region as an epidemiological surveillance tool. Several studies that have focused on candidemia demonstrate new resistant and emerging species (14,15). Brazil is one of the most populated countries in the world and presents a remarkable variety of regions in distinct stages of development. São Paulo is the most developed state and has the highest number of hospitals (>800) in the country, but few data on the causative agents of candidemia are available (3,16–18). The comprehensive regional distribution of causative species and antifungal susceptibility patterns provides support to surveillance programs at both the local and national levels and has a positive impact on patient survival (19).

The relative distribution of species causing candidemia can vary according to hospital characteristics (20). In this study, we tested isolates obtained from public hospitals, locally identified as non-*C. albicans*, and although there are some limitations in diagnosis due to deficiency of resources in these hospitals (21), in such settings, a large proportion (40.9%) were teaching hospitals.

We verified *C. parapsilosis* s.s. as the prevalent species (31.9%), followed by *C. albicans* (27.8%). The relevance of *C. parapsilosis* s.s. in Brazilian candidemia cases has been demonstrated in previous studies (22,23) and seems to be associated with cases identified at public hospitals (24 [Bibr B25]).

*Candida glabrata* was previously recognized as an emerging species in some Brazilian health centers (17,18,21,24). We also noted this trend in our isolates from the Southeast part of Brazil with *C. glabrata* ranking fourth, accounting for 9.7% of candidemia cases. In the Brazilian literature, few published studies have *C. glabrata* prevalence rates as high as 8% (18,24–27). *Candida glabrata* is recognized as an emerging species in Brazil, similar to what has been reported in some countries in Europe, Asia, and South America while it has already emerged in the United States as the second most prevalent species (28–31). The percentage of VCZ non-wild type *C. glabrata* isolates was high (28.6%) in our study. These isolates were often FCZ-resistant as well, one of the highest rates among Brazilian studies (22,24,32). We observed no resistance for echinocandins in *C. glabrata*. Only ∼50% of Latin American hospitals have echinocandins available for candidemia treatment, thus azoles resistance characterizes a serious health problem (33).

The frequency of *C. guilliermondii* s.s (2%) verified in this study is similar to what has been previously reported in Brazil (18,20). The largest candidemia study performed in South and Central America showed a 6.5% prevalence of *C. guilliermondii* s.s on the continent and an incredibly high burden (20.7%) in Honduras (26). There was one FCZ- and VCZ-resistant non-wild type *C. guilliermondii* s.s strain but the strain and the other isolate were susceptible or wild type to all other antifungals tested. As previously described in Brazil, our *C. guilliermondii* s.s and *C. glabrata* isolates were more susceptible to FCZ than reported in other countries (20,34).

The relative rate of *C. haemulonii* complex isolates was high (4.2%) compared to previous studies showing frequencies <2% in Brazil (35,36), Asia (14,37), South America (26), and several countries as published by SENTRY antifungal surveillance (15). A higher frequency (15%) of *C. haemulonii* complex was reported only in India (38), but there was no mention of usage of molecular tools essential for ascertaining the frequency of cryptic species. In our study, we did not find high MICs for *C. haemulonii* s.s., however all *C. haemulonii* var. *vulnera* showed elevated FCZ MICs. *Candida duobushaemulonii* was unique in that it showed high MICs for AmB, and in an earlier report in Sao Paulo State (35,36), this species was cited as having the highest MIC values to polyenes in the complex as well as presenting elevated MICs to fluconazole (36). The lack of clinical breakpoints for this species limits the ability to characterize resistance, however, *C. duobushaemulonii* has a large potential to multidrug resistance. VCZ MICs were not elevated, which differs from other studies (14,39). A recent Brazilian study has shown that resistance to oxidative stress along with changes in homeostasis, mitochondrial function, and ergosterol may be related to AmB resistance in the *C. haemulonii* complex (40). Cases of C. *haemulonii* complex are increasing according to recent reports, and more research is needed to understand antifungal susceptibility within the complex (36). *C. kefyr* is a rare cause of candidemia (>1%), and the single isolate in our study was non-wild type for both VCZ and CAS.

This study has some limitations. All bloodstream isolates are expected, but not compulsory, to be sent to the State Reference Laboratory, but some *Candida* isolates from the hospitals under surveillance may have been left out of the study. Our study covered isolates from 22 hospitals and, although it encompassed a great variety of hospitals, it may not be representative of the entire country. Even if this study comprises only a fraction of the large geographic and demographic territory of Brazil, our findings may still add useful etiologic data to the global surveillance of candidemia.

We showed that although *C. albicans* s.s. and *C. parapsilosis* s.s. remain the most frequent species in São Paulo state, *C. haemulonii* complex was verified to be a frequent causative agent of candidemia in the state. Moreover, as *C. haemulonii* complex is easily misidentified as *C. auris*, a multidrug-resistant and emerging species, accurate characterization of candidemia species is justified. Additionally, we highlight the emergence of *C. glabrata* in Brazil and, notably, the significant number of strains associated with high MICs to both FCZ and VCZ. The multidrug-resistant species of *C. guilliermondii* complex are still rare in our region.

Finally, many common *Candida* species are now considered species complexes and the identification of cryptic species is problematic but of clinical relevance since they may have different infection sites, infection severity, and distinct antifungal susceptibility patterns. Although there is no consensus about the need to routinely differentiate sibling species in the clinical laboratory, strategies such as a laboratory network for centralizing molecular testing in big reference centers, as occurs in Brazil, contributes significantly to describing epidemiological data such as species distribution and antifungal susceptibility profile in geographical regions.

## Supplementary Material

Click here to view [pdf].
